# Metabolic Response of Visceral White Adipose Tissue of Obese Mice Exposed for 5 Days to Human Room Temperature Compared to Mouse Thermoneutrality

**DOI:** 10.3389/fphys.2017.00179

**Published:** 2017-03-23

**Authors:** Inge van der Stelt, Femke Hoevenaars, Jitka Široká, Lidwien de Ronde, David Friedecký, Jaap Keijer, Evert van Schothorst

**Affiliations:** ^1^Human and Animal Physiology, Wageningen UniversityWageningen, Netherlands; ^2^Laboratory of Metabolomics, Institute of Molecular and Translational Medicine, Faculty of Medicine and Dentistry, Palacky University OlomoucOlomouc, Czechia

**Keywords:** indirect calorimetry, serum metabolomics, thermogenesis, thermoneutrality, transcriptomics, visceral white adipose tissue

## Abstract

Housing of laboratory mice at room temperature (22°C) might be considered a constant cold stress, which induces a thermogenic program in brown adipose tissue (BAT). However, the early adaptive response of white adipose tissue (WAT), the fat storage organ of the body, to a change from thermoneutrality to room temperature is not known. This was investigated here for various WAT depots, focusing on epididymal WAT (eWAT), widely used as reference depot. Male adult diet-induced obese (DIO) C57BL/6JOlaHsd mice housed at thermoneutrality (29°C), were for 5 days either switched to room temperature (22°C) or remained at thermoneutrality. Energy metabolism was continuously measured using indirect calorimetry. At the end of the study, serum metabolomics and WAT transcriptomics were performed. We confirmed activation of the thermogenic program in 22°C housed mice. Body weight and total fat mass were reduced. Whole body energy expenditure (EE) was increased, with a higher fatty acid to carbohydrate oxidation ratio and increased serum acylcarnitine levels, while energy intake was not significantly different between the two groups. Transcriptome analysis of eWAT identified tissue remodeling and inflammation as the most affected processes. Expression of pro-inflammatory M1 macrophage-related genes, and M1 over M2 macrophage ratio were decreased, which might be linked to an increased insulin sensitivity. Markers of thermogenesis were not altered in eWAT. Decreased expression of tryptophan hydroxylase 2 (*Tph2*) and cholecystokinin (*Cck*) might represent altered neuroendocrine signaling. eWAT itself does not show increased fatty acid oxidation. The three measured WATs, epididymal, mesenteric, and retroperitoneal, showed mainly similar responses; reduced inflammation (s100a8), decreased carbohydrate oxidation, and no or small differences in fatty acid oxidation. However, Ucp1 was only expressed and increased in rWAT in 22°C housed mice. Cck expression was decreased in the three WATs, significantly in eWAT and rWAT, in contrast to Tph2, which was decreased in eWAT while not expressed in mWAT and rWAT. Our data show that tissue remodeling, inflammation and neuroendocrine signaling are early responses in WAT to a moderate decrease in environmental temperature.

## Introduction

Lowering ambient temperature, to temperatures below the thermoneutral zone of endotherm animals results in thermogenesis. In this process energy is used to produce heat for maintenance of body temperature. In an acute cold exposure, this is achieved by the combination of shivering thermogenesis—heat production resulting from muscle contractions—and non-shivering thermogenesis where energy is dissipated mainly in brown adipose tissue (BAT), and to a lesser extent via increased futile cycling.

The process of non-shivering thermogenesis starts when cold activates thermoreceptors in peripheral tissues, which triggers the sympathetic nervous system (SNS). This results in increased noradrenaline levels at nerve endings of target tissues, which activates local β-adrenergic receptors (Hsieh and Carlson, 1957; Ye et al., 2013). In BAT, generation of cyclic adenosine monophosphate (cAMP) results in lipolysis. Free fatty acids (FFAs) activate uncoupling protein 1 (UCP1), overriding inhibition by GDP and ADP. UCP1, which is embedded in the mitochondrial inner membrane, uncouples ATP production from the respiratory chain, thereby producing heat but no ATP (Heaton et al., 1978, for an extended overview see Cannon and Nedergaard, 2004). Beta-adrenergic activation, e.g., by cold exposure, also increases the transcription of *Ucp1*. Extended cold exposure will increase mitochondrial biogenesis as well as hyperplasia and hypertrophy of BAT (Lowell and Spiegelman, 2000).

In recent years, it has become clear that differences in ambient temperatures can have large effects on metabolism, throughout the body. Laboratory animals, such as mice, are routinely housed at room temperature (~22°C), which is below the lower critical temperature (~28°C) of their thermoneutral zone. Housing mice at room temperature elevated heart rate up to 100% (Swoap et al., 2008), and increased basal metabolic rate by 50–100% (Golozoubova et al., 2004) compared to mice housed at thermoneutral conditions. Mice housed at temperature below their thermoneutral zone might therefore be considered to be under constant cold stress. This impacts research outcomes, which can be different and even conflicting when conducting experiments at either room temperature or thermoneutrality. For example, UCP1 knockout mice were expected to become obese, especially when feeding a high-fat diet. However, this was not observed at room temperature (Enerback et al., 1997). Later, it was shown that housing these mice at 30°C did induce obesity (Feldmann et al., 2009). These and other studies have provided grounds for discussion about best housing conditions of laboratory mice in relation to translatability to the human situation (Overton, 2010; Cannon and Nedergaard, 2011; Speakman and Keijer, 2012). Above all, insight in the adaptive responses of metabolic tissues to changes in environmental temperature is needed for improved interpretation and translation of metabolic studies.

Although the role of BAT in non-shivering thermogenesis is evident, the attribution of white adipose tissue (WAT) to this process is less defined. WAT is the body's fat storage organ, with an important endocrine function (Wang et al., 2008). Upon cold exposure, WAT is important for whole body metabolism, as WAT is thought to be the main provider of energy in the form of FFAs, which are subsequently oxidized in BAT triggering UCP1 activation (Karp, 2012; Bartelt and Heeren, 2014). Activated WAT lipolysis is the result of noradrenalin released from the active SNS acting on β-adrenergic receptors in WAT. Increasing sympathetic tone as a result of cold exposure also increases WAT vascularization (Xue et al., 2009). Decreasing this SNS signaling by surgical denervation increased WAT mass and adipocyte number (Bowers et al., 2004), further establishing the link between SNS signaling and WAT lipolysis. While BAT may be a major player in the regulation of thermogenesis, research focussing on the effects of WAT adaptation to cold stress is limited.

In summary, while the adaptation of BAT to cold stress is well understood, less is known about WAT despite its important role in metabolism. We focussed on visceral WAT, instead of subcutaneous WAT, as visceral fat depots are more responsive to adrenergic-dependent lipolysis, are more active in metabolic processes and are closer linked to health risks as a result of obesity (Tran et al., 2008; Ibrahim, 2010). In addition, epididymal WAT (eWAT) is the widest metabolically investigated WAT in rodents. To better understand the early-phase adaptive response of eWAT to cool stress, we compared mice that were transferred from thermoneutrality (29°C) to room temperature (22°C) with mice continuing at thermoneutrality (29°C). We fed the mice a human-relevant diet. After a short term adaptation of 5 days, we expected adaptive changes measurable at all levels (Bartelt and Heeren, 2014). We measured whole body energy balance, serum metabolites and performed whole genome gene expression analysis of eWAT to delineate the short term changes induced by a reduction in housing temperature in DIO mice, focusing on metabolism of eWAT. Responses were subsequently verified and compared with WAT depots of mesenteric and retroperitoneal origin using a subset of genes.

## Materials and methods

### Animal study

The animal experiment was performed according to the Dutch animal experimentation act (1996). Permission for this study was granted by the Animal Ethical Committee of Wageningen University (DEC 2012056).

Male wild-type C57BL/6JOlaHsd mice (Harlan Laboratories, Horst, The Netherlands), 9 weeks of age, were housed at thermoneutral temperature (29–30°C) under environmentally controlled conditions (12 h light/dark cycle, 55 ±15% humidity). The mice had *ad libitum* access to food and water, which were weekly renewed. During the first 3 weeks, the mice were acclimatized in which they received a purified low fat diet (LFD; 3,865 kcal/kg, 10 energy% fat), followed by a human-relevant purified high fat diet (HFD; 4,700 kcal/kg, 40 energy% fat); dietary compositions are according to Hoevenaars et al. (2012) and Voigt et al. (2013). The diets were produced by Research Diet Services (Wijk bij Duurstede, The Netherlands). After 12 weeks of HFD feeding, fat mass of the mice was above the cut-off value of 25%, which is used in human studies to diagnose obesity (Okorodudu et al., 2010). The mice were stratified on fat mass at this point into an experimental group (*n* = 12) switched from thermoneutral housing temperature to room temperature (22°C) for 5 days, during which they remained in the indirect calorimetry system, and a control group remaining at thermoneutrality (*n* = 24), of which half (*n* = 12) was used for reference measurements during 48 h in the indirect calorimetry system. Body weight and body composition were measured before and at the end of the five experimental days (EchoMRI 100V, EchoMedical Systems, Houston, TX, USA). Adiposity was calculated as percentage of fat mass over body weight. Food intake was measured in the indirect calorimetry system continuously. Feces of the mice were collected quantitatively before, at 29°C housing, and after 5 days of 22°C housing (same mice). Energy content (*n* = 12) was analyzed in collected feces by bomb calorimetry (IKA C7000, IKA Works, Staufen, Germany). All samples were measured in duplicate. Energy loss (kJ/day) was calculated as amount of feces (g/day) ^*^ fecal energy content (kJ/g).

### Indirect calorimetry

Indirect calorimetry (*n* = 12) was performed using an open circuit LabMaster Metabolism Research Platform (TSE systems GmbH, Bad Homburg, Germany), as described (Duivenvoorde et al., 2015). The data of the VCO_2_ and VO_2_ of the last 24 h in indirect calorimetry was used for mice housed at either 29°C or 22°C. Respiratory exchange ratio (RER) is defined as VCO_2_ divided by VO_2_, and energy expenditure (EE, kcal/hr) was calculated with TSE Software 4.2.3 using the equation EE = [3.941 × VO_2_ + 1.106 × VCO_2_]/1,000.

During indirect calorimetry, locomotor activity of mice (*n* = 8) was continuously measured with infrared beams in horizontal x- and y-directions (ActiMot system, TSE systems). Physical activity is expressed as total (ambulatory plus fine movements) light beam breaks. Measurements of the last 24 h were used for analysis.

### Blood and tissue collection

Mice were sacrificed using decapitation after a 2 h food deprivation in the light phase. Whole blood glucose levels (*n* = 12) were measured using the ADC Freestyle Lite (Abbott B.V., Zwolle, the Netherlands). Whole blood hematocrit levels (*n* = 12) were determined by using 60 μl heparinized capillary tubes (Hirschmann Laborgeräte, Eberstadt, Germany), which were centrifuged in a micro-hematocrit centrifuge at 3,000 × g for 5 min. Serum tubes (Greiner Bio-one, Longwood, USA) were used to extract serum from whole blood; the serum was aliquoted and stored at −80°C.

Epididymal white adipose tissue (eWAT) from the left fat pad, mesenteric WAT around the intestines (mWAT), retroperitoneal WAT at the back of the kidneys (rWAT), and interscapular BAT were collected, snap frozen into liquid nitrogen and stored at −80°C.

### Serum parameters

Serum leptin, adiponectin, insulin and glucagon levels (*n* = 8) were measured in duplicate using the Bio-Plex Pro mouse diabetes assay (Bio-Rad laboratories, Veenendaal, the Netherlands) according to the manufacturer's instructions. Samples were diluted 25x for measurement of leptin, insulin and glucagon, and 1,600x for adiponectin. HOMA-IR was calculated as fasting serum insulin (pmol/l) ^*^ fasting plasma glucose (mmol/l)/135 (Meyer et al., 2006).

Serum FFA (*n* = 12) were measured in duplicate using a NEFA-HR kit (Wako chemical GmbH, Neuss, Germany) according to the manufacturer's instructions. Serum triglycerides and glycerol (*n* = 12) were measured in triplicate using a triglyceride liquicolor mono kit (Human Diagnostics, Wiesbaden, Germany) and glycerol colorimetric kit (Instruchemie BV, Delfzijl, The Netherlands), respectively, according to the manufacturer's instructions. Serum triglyceride levels were corrected for free glycerol levels (*n* = 12) by subtraction.

For metabolome analysis, 3 μl of serum (*n* = 10) was mixed with 97 μl of methanol solution containing 0.1% formic acid and deuterated internal standards (amino acids and acylcarnitines, non-derivatized kit, Chromsystems, Munich, Germany). Final solutions were vortexed and stored overnight at −80°C. After centrifugation (10 min, 24,400 g, 4°C), 80 μl of supernatant was used for flow injection mass spectrometry analysis. Remaining supernatant was pooled and used for quality control purposes.

The samples were measured on a 5,500 QTrap (AB Sciex, Foster City, CA, USA) as published (Janeckova et al., 2012) with modifications. Settings as follows: polarity was set to positive mode with ionspray voltage of 5,500 V, capillary temperature of 450°C, curtain gas of 25 psi, ion source gas (GS1/GS2) of 35 psi. Methanol containing 0.1% formic acid was chosen as a mobile phase. Flow rate was set at 0.05 mL/min (0.0–1.0 min) and 0.30 mL/min (1.0–1.5 min). Compounds were measured in multiple reaction monitoring mode under optimized parameters of declustering potential and collision energy for each mass transition. Unit resolution was set for isolation ions in mass analyzer. Data were processed by software Chemoview 2.0 (AB Sciex).

### RNA isolation

Total RNA was isolated from BAT and WAT depots as published (Van Schothorst et al., 2005), followed by a clean-up using RNeasy mini kit (Qiagen GmbH, Hilden, Germany). RNA yield and purity were checked using a Nanodrop spectrophotometer (IsoGen Life Science, Maarsen, The Netherlands) and RNA quality was verified with the Experion automated electrophoresis system (Bio-Rad), using Experion StdSens chips (Bio-Rad).

### Reverse-transcription quantitative real-time polymerase chain reaction

Reverse-transcription quantitative real-time polymerase chain reaction (RT-qPCR) was performed as previously described (Hoek-van den Hil et al., 2013). Individual samples were measured in duplicate. Standard curves of pooled samples, negative controls, melting profiles, *R*^2^ and PCR efficiency were used for validation of each run according to the MIQE guidelines (Bustin et al., 2009). Gene expression of the following genes was analyzed in BAT: fibroblast growth factor 21 (*Fgf21*), peroxisome proliferative activated receptor, gamma, coactivator 1 alpha (*Ppargc1a*), and uncoupling protein 1 (*Ucp1*) using reference genes beta-2 microglobin (*B2m*) and calnexin (*Canx*) for normalization. In WAT, mRNA expression of a number of genes including cholecystokinin (*Cck*) and tryptophan hydroxylase (*Tph2*) was analyzed, using reference genes *B2m* and ribosomal protein S15 (*Rps15*). To measure the low *Tph2* expression, 2 μl of cDNA was pre-amplified for 20 cycles with the addition of 23 μl SsoAdvanced Preamp supermix (Bio-Rad) combined with *Tph2* primers according to the manufacturer's protocol. An overview of all genes, primers and their sequences used to analyse BAT and three WAT depots can be found in Supplementary Table 1. Relative gene expression was expressed as the normalized expression values compared to the mean of the values of the 29°C housed control mice set at 1.

### Whole genome gene expression microarrays

RNA (200 ng) from eWAT (*n* = 10) was used as input material for whole mouse genome 8^*^60K microarrays (G4852A, Agilent Technologies, Santa Clara, CA, USA). Arrays were conducted according to the manufacturer's protocol with a few modifications as described previously (van Schothorst et al., 2007). Quantification of signals was performed using Feature Extraction version 10.7.3.1 (Agilent), followed by quality control and normalization as published (Hoek-van den Hil et al., 2013). Microarray data have been deposited in NCBI Gene Expression Omnibus (GEO) under accession number GSE53805. Of the 30,733 probes that were considered expressed (51.5% of total probes), 142 probes were considered significantly different as analyzed by a Student's *t*-test using Benjamini-Hochberg false discovery rate adjustments (FDR, *p* < 0.05). Fold change is expressed as ratio of the expression of the 22°C housed mice over 29°C housed control mice. Analysis of process networks and GO processes of all genes with a FDR-adjusted *p* < 0.1 was performed using Metacore (Thomson Reuters, New York, NY, USA). Next, significantly different expressed genes with known functions (93 genes) were assigned manually to biological processes using recent literature and gene function databases. Expression of known, literature based, key genes involved in these biological processes was added to the analysis to check involvement of these processes in the 22°C housed mice.

### Western blotting

Total eWAT protein was extracted by homogenizing eWAT in 2 μl assay buffer per mg tissue using a motorized pestle. Hereafter, samples were sonicated for 18 pulses at 40% amplitude using a SLPe sonicator (Branson, Danbury, CT, USA). Samples were centrifuged for 10 min at 18,620 × g.

Samples were heated for 5 min at 95°C and 3 μl of protein per sample was run on a 14% Tris-tricine gel, after which the protein was transferred to an Immobilon PVDF membrane (Merck Millipore, Amsterdam, the Netherlands). The membrane was blocked for 1 h with 1:1 Odyssey blocking buffer (LI-COR, Lincoln, NE, USA) and TBS. The membrane was incubated with 1:200 diluted goat anti-CCK (sc-21617, Santa Cruz Biotechnology, Santa Cruz, CA, USA.) for 48 h at 4°C in 1:1 Odyssey blocking buffer and TBS with 0.05% Tween20. After washing with TBS plus 0.1% Tween20 (TBST), the membrane was incubated with 1:5,000 diluted donkey anti-goat (926-32214, LI-COR) for 1 h at room temperature in 1:1 Odyssey blocking buffer and TBST. After washing with TBST, the membrane was scanned on an Odyssey scanner (LI-COR). As loading control, the membrane was incubated with 1:2,000 rabbit anti-β-Actin (ab8227, Abcam, Cambridge, MA, USA) and 1:5,000 donkey anti-rabbit (926-32223, LI-COR). Bands were analyzed using Image studio Lite (LI-COR), and relative CCK protein levels were normalized using β-Actin signal, after which average control group level was set at 1.

### Statistical analysis

Graphpad Prism version 5.04 (Graphpad Software, San Diego, CA, USA) was used for statistical analysis. Comparisons within animals before and after the 5-day period of lower housing temperature were done using a paired Student's *t*-test (body weight, body composition, adiposity and fecal energy loss), for measurements over time one-way ANOVA was used with Dunnett's *post-hoc* test (food intake). Unpaired Student's *t*-tests were performed to compare means of 22°C and 29°C groups for normally distributed data (either with or without log transformation). For non-normally distributed data Mann-Whitney U test was used. Differences were considered significant for *p* < 0.05. Data is presented as mean ± SEM.

## Results

### Thermogenesis in BAT

**Figure 1:**
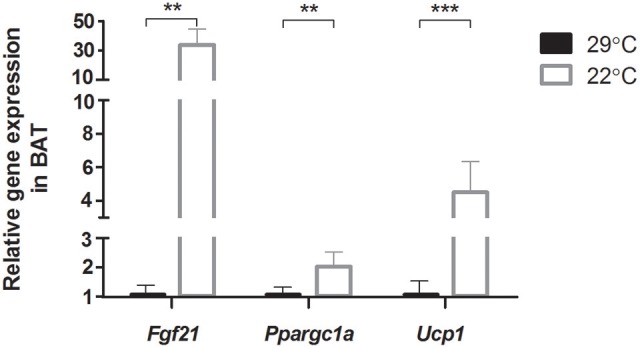
**Relative gene expression of genes involved in adaptive thermogenesis in brown adipose tissue (BAT)**. Thermoneutral housed mice were switched to 22°C for 5 days (*n* = 12) vs. control thermoneutral (29°C) housed mice. Full gene names are listed in Supplementary Table 1. Data is presented as mean ± SEM (*n* = 7–11). Significant different values of the 22°C housed mice compared to the 29°C housed mice are indicated with ^**^p < 0.01, and ^***^p < 0.001.

Gene expression levels of *Fgf21, Ppargc1a*, and *Ucp1* in BAT were upregulated in mice that were switched to room temperature (22°C) for 5 days, compared to control mice that were kept at thermoneutral temperature (29°C) (Figure 1). This confirms the activation of the thermogenic program in BAT in mice that were switched to the cooler environment.

### Body composition and indirect calorimetry

**Figure 2:**
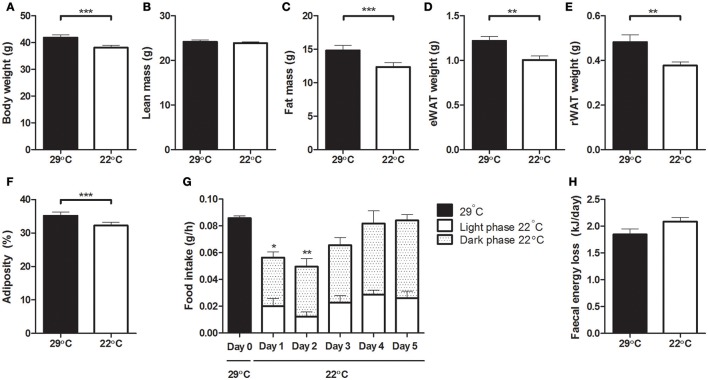
**Body weight, body composition, food intake and fecal energy loss at 29°C or after switching to 22°C**. **(A)** Body weight, **(B)** total lean mass, **(C)** total fat mass, **(D)** epididymal white adipose tissue weight, **(E)** retroperitoneal white adipose tissue weight, **(F)** adiposity; fat mass as percentage of body weight, **(G)** food intake, averaged per hour and differentiated between the inactive light phase and active dark phase, **(H)** fecal energy loss. All data was collected in the same set of mice, before and after the temperature switch. Data is presented as mean ± SEM (*n* = 12). Significant different values of the 22°C housed mice compared to the control 29°C housed mice are indicated with ^*^p < 0.05, ^**^p < 0.01, and ^***^p < 0.001.

Mice that were switched to 22°C showed a significantly lower body weight compared to their weight at 29°C (Figure 2A). On average, these cooler housed mice lost 3.81 ± 0.38 grams of bodyweight, while mice remaining at 29°C gained 0.70 ± 0.13 grams of bodyweight during the last 5 days of the experiment. As lean mass remained unaffected (Figure 2B), this decrease in body weight in the 22°C housed mice was due to a decrease in fat mass (Figure 2C), which was relatively similar decreased in both eWAT and rWAT depots (Figures 2D,E), thus decreasing adiposity (Figure 2F). Upon the change to 22°C, food intake decreased immediately, mainly during the active dark phase. However, after 2 days, food intake was not significantly different from intake at the start of the experimental period, i.e., before the temperature change (Figure 2G). Fecal energy loss was not significantly different before and after changing the ambient temperature (Figure 2H).

**Figure 3:**
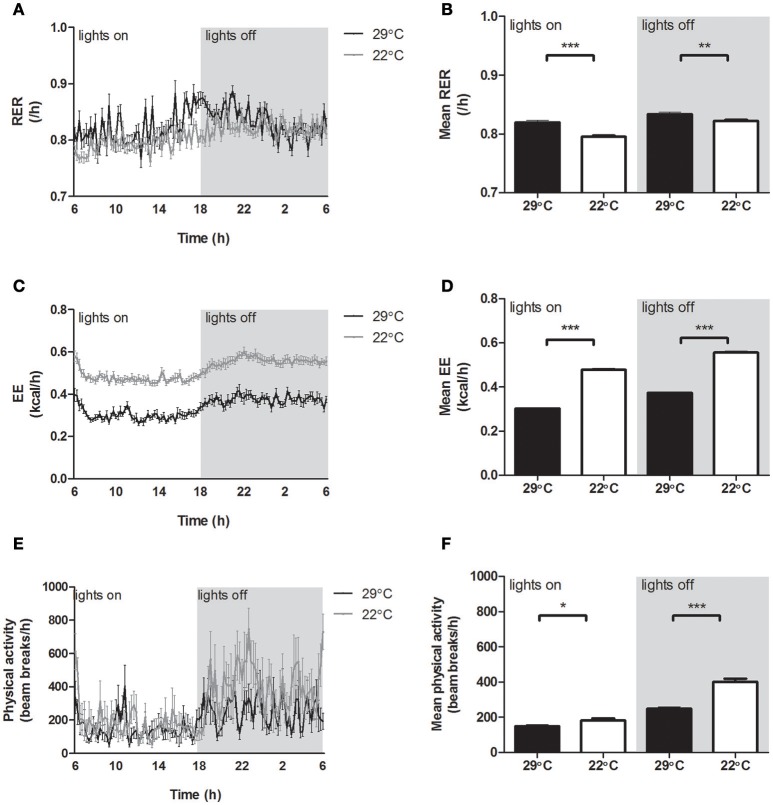
**Energy balance of mice housed at 29°C or 22°C. (A)** 24 h Respiratory exchange ratio (RER), **(B)** average RER, **(C)** 24 h energy expenditure (EE), **(D)** average EE, **(E)** 24 h physical activity (*n* = 8), **(F)** average physical activity (*n* = 8) for mice fed a high fat diet housed at thermoneutrality (black) or 22°C (gray lines, white bars). Values are displayed for both inactive light phase and active dark phase (gray area). Data shown is calculated from the last 24 h in the indirect calorimetry system and is presented as mean ± SEM (*n* = 12), unless stated otherwise. Significant different values of the 22°C housed mice compared to the 29°C housed mice are indicated with ^*^p < 0.05, ^**^p < 0.01, and ^***^p < 0.001.

Mice housed at 22°C showed a significantly lower RER compared to control mice (Figures 3A,B), indicating a switch in metabolism toward increased fatty acid oxidation and reduced carbohydrate oxidation. Energy expenditure (EE) was continuously increased in the mice housed at 22°C (Figures 3C,D), partly due to significantly increased physical activity in mice housed at 22°C, which was most pronounced in the dark phase (Figures 3E,F).

### Circulating metabolites

Several circulating metabolites were measured to assess energy metabolism and health status. Mice housed at 22°C showed significantly lower blood glucose levels, higher hematocrit levels, impressively decreased serum insulin and leptin levels, and a decreased HOMA-IR (Table 1). Furthermore, most (acyl)carnitine levels were significantly increased, however, C0 (free carnitine) and C3 levels were significantly decreased, supporting increased whole body fat oxidation. Similar to the overall increase in acylcarnitines, a trend toward increased FFA levels was seen. Serum triacylglycerides (TG) levels were significantly lower at 22°C. Lastly, the lower ambient temperature significantly decreased the majority of serum amino acids concentrations compared to control mice (Table 1).

**Table 1:** **Circulating metabolites of mice housed at 22°C or at control 29°C for 5 days**.

	**29°C**	**22°C**		
Glucose (mmol/l)	6.46 ± 0.21	5.88 ± 0.14	^*^	Down
Hematocrit (%)	46.96 ± 0.58	49.68 ± 0.77	^*^	Up
Adiponectin (μg/ml)	9.85 ± 0.50	8.62 ± 0.85		
Glucagon (ng/ml)	0.80 ± 0.06	0.70 ± 0.07		
Insulin (ng/ml)	15.10 ± 1.83	7.24 ± 1.25	^**^	Down
HOMA-IR (pmol/l^*^mmol/l)	127.61 ± 16.44	54.35 ± 9.23	^**^	Down
Leptin (ng/ml)	118.89 ± 1.83	31.77 ± 5.06	^**^	Down
FFA (μmol/l)	617.31 ± 34.80	721.95 ± 38.72	*P* = 0.0811	
TG (mg/mg protein)	90.37 ± 6.85	48.97 ± 3.08	^***^	Down
**CARNITINES (μmol/l)**				
C0	20.30 ± 1.01	14.53 ± 0.52	^***^	Down
C2	10.12 ± 0.50	14.83 ± 1.01	^***^	Up
C3	0.192 ± 0.013	0.159 ± 0.006	^*^	Down
C14	0.052 ± 0.003	0.048 ± 0.001		
C14:1	0.063 ± 0.004	0.066 ± 0.002		
C14:2	0.018 ± 0.001	0.022 ± 0.0.01	^*^	Up
C16	0.143 ± 0.007	0.162 ± 0.007	^*^	Up
C16:1	0.052 ± 0.004	0.050 ± 0.002		
C18	0.041 ± 0.002	0.049 ± 0.003	^*^	Up
C18:1	0.185 ± 0.011	0.236 ± 0.013	^**^	Up
C18:2	0.109 ± 0.005	0.155 ± 0.006	^***^	Up
**AMINO ACIDS (μmol/l)**				
Alanine	488.27 ± 38.80	421.65 ± 17.85		
Arginine	242.68 ± 35.95	115.99 ± 30.04	^*^	Down
Citrulline	66.27 ± 3.27	44.75 ± 2.33	^***^	Down
Glutamic acid	99.06 ± 14.85	79.05 ± 5.96		
Glycine	333.31 ± 13.91	288.61 ± 15.26	^*^	Down
Histidine	47.27 ± 3.13	36.76 ± 1.35	^**^	Down
Lysine	393.76 ± 26.12	297.27 ± 20.79	^**^	Down
Methionine	67.11 ± 7.23	44.70 ± 4.13	^*^	Down
Ornithine	116.84 ± 12.79	78.50 ± 7.87	^*^	Down
Phenylalanine	101.11 ± 8.95	68.92 ± 4.36	^**^	Down
Proline	108.64 ± 8.99	81.84 ± 7.97	^*^	Down
Threonine	23.96 ± 1.80	19.46 ± 1.05	^*^	Down
Tryptophan	42.88 ± 2.44	31.55 ± 1.82	^**^	Down
Tyrosine	111.17 ± 10.16	76.26 ± 6.99	^*^	Down
Valine	139.69 ± 10.89	111.42 ± 6.64	^*^	Down
(Iso)leucine	309.27 ± 34.81	211.29 ± 14.24	^*^	Down

Glucose and hematocrit levels were measured in blood. Adiponectin, glucagon, insulin, and leptin concentrations (all n = 8), free fatty acid (FFA), and triglycerides (TG) concentrations (both n = 12), carnitine and amino acid concentrations (both n = 10) were measured in serum. HOMA-IR was calculated using insulin and glucose levels (n = 8). Temperatures indicate housing temperatures of the mice. Lower or higher significant concentrations at 22°C are indicated with down or up respectively. Data is presented as mean ± SEM. Significant different values are indicated with

* *p < 0.05*,

** p < 0.01, and

*** *p < 0.001*.

### WAT transcriptomics

**Figure 4:**
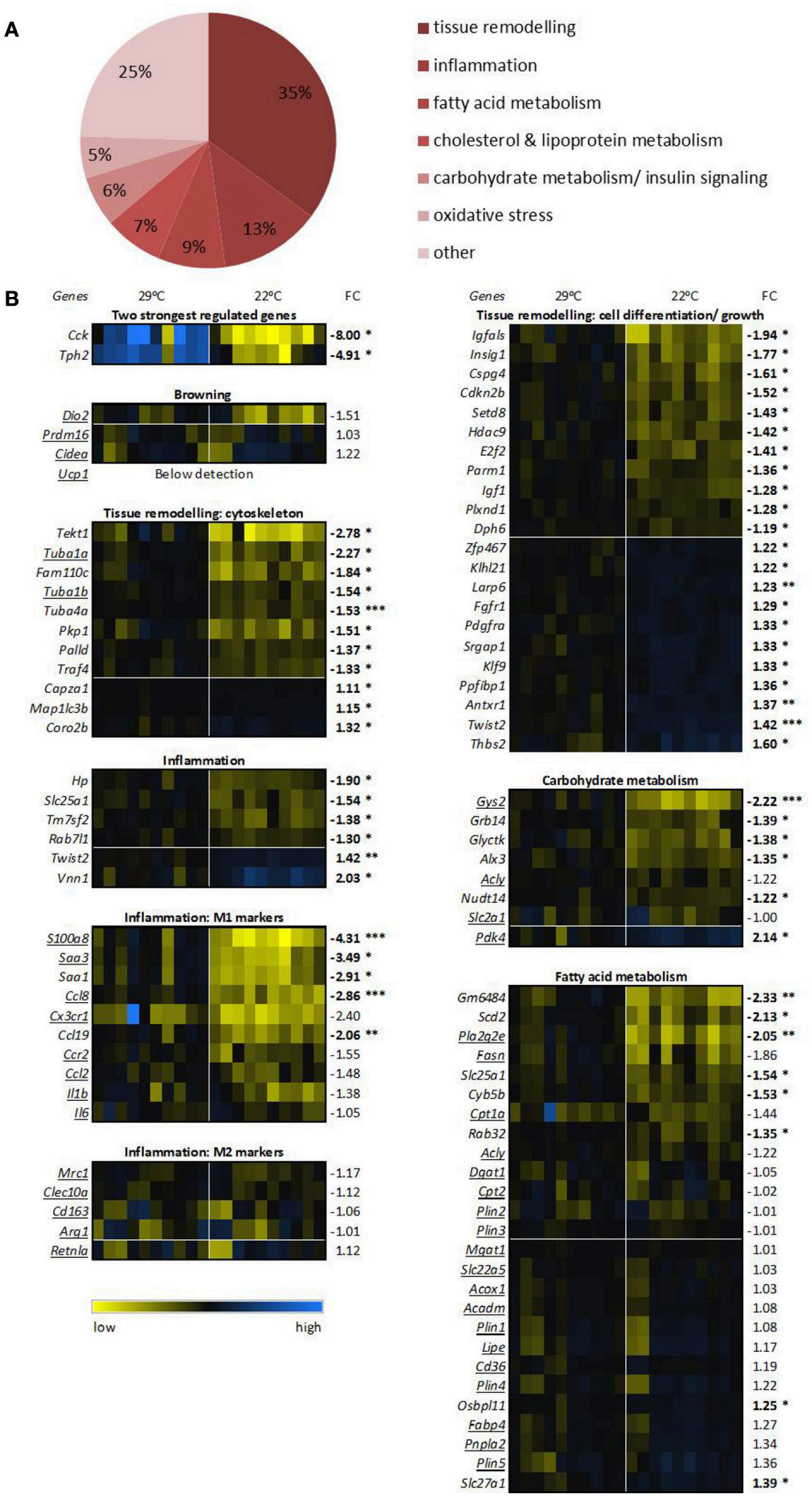
**Transcriptomics of epididymal white adipose tissue. (A)** Differential expression of processes between 29°C and 22°C housed mice (*n* = 10). Processes are ranked based on known functions of all genes with significant false discovery rate adjusted *p*-values. **(B)** Expression of key genes for the top 2 differential expressed processes, processes involved in metabolism, and the 2 genes with the highest fold change (FC). Expression of the control 29°C group has been set at 1. Underlined genes are key markers for the assigned processes. Genes are ranked based on FC, bold numbers indicate significantly different transcript levels between the 22°C vs. control 29°C housed mice and are assigned with ^*^p < 0.05, ^**^p < 0.01, and ^***^p < 0.001. See Supplementary Table 2 for gene names, systematic names, and full *p*-values.

**Figure 5:**
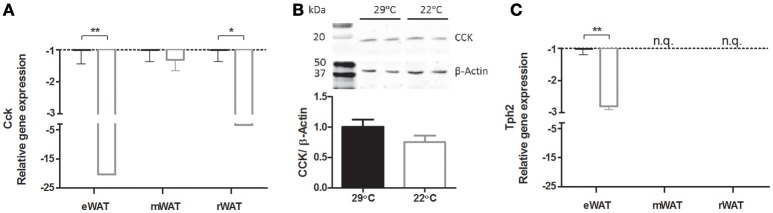
**Relative expression of *Cck* and *Tph2* in white adipose tissues at 29°C or 22°C ambient temperature. (A)** *Cck* gene expression in white adipose tissue of epididymal (eWAT), mesenteric (mWAT), and retroperitoneal (rWAT) origin, as measured by RT-qPCR (*n* = 7–10), **(B)** Representative samples of CCK protein levels in eWAT as measured by Western blot and normalized using β-Actin (*n* = 11–12), **(C)** *Tph2* gene expression as measured by RT-qPCR (*n* = 8–10). Cck: cholecystokinin; *Tph2*, tryptophan hydroxylase 2. Data is presented as mean ± SEM. N.q., not quantifiable. Significant different values of the 22°C housed mice (white bar) compared to the control 29°C housed mice (black bar) are indicated with ^*^p < 0.05, ^**^p < 0.01.

Next, global gene expression in eWAT of mice switched to 22°C vs. continuous 29°C was analyzed. In total, 125 unique genes (142 probes) were differentially regulated between the two groups of mice. The top 20 up regulated and down regulated unique genes can be found in Table 2. The two genes with the highest absolute fold change of 22°C vs. 29°C housing were cholecystokinin (*Cck*, fold change of −8.00) and tryptophan hydroxylase 2 (*Tph2*, fold change of −4.91) (Figure 4B). The massively decreased *Cck* expression in 22°C housed mice was confirmed by RT-qPCR, and was also observed, albeit to a lesser extent, in rWAT, but not in mWAT (Figure 5A). eWAT CCK protein levels showed a 25% decrease, although non-significant, in mice housed at 22°C (Figure 5B). While RT-qPCR readily confirmed the strongly decreased *Tph2* expression in eWAT of 22°C housed mice, *Tph2* levels in mWAT and rWAT remained below quantification levels (Figure 5C).

**Table 2:** **Top 20 up and down regulated unique genes (FDR *p* < 0.05) of epididymal WAT**.

**Gene symbol**	**Gene name**	**Systematic name**	**Fold change**	**Assigned process**
Pdk4	Pyruvate dehydrogenase kinase, isoenzyme 4	NM_013743	2.14	Carbohydrate metabolism/insulin signaling
Vnn1	Vanin 1	NM_011704	2.03	Inflammation, oxidative stress
Thbs2	Thrombospondin 2	NM_011581	1.60	Tissue remodeling
Tmem140	Trans membrane protein 140	NM_197986	1.52	Unknown function
Txnip	Thioredoxin interacting protein	NM_001009935	1.49	Oxidative stress
Twist2	Twist basic helix-loop-helix transcription factor 2	NM_007855	1.42	Inflammation, tissue remodeling
F3	Coagulation factor III	NM_010171	1.42	Other
Slc27a1	Solute carrier family 27 (fatty acid transporter), member 1	NM_011977	1.39	Fatty acid metabolism
Antxr1	Anthrax toxin receptor 1	NM_054041	1.37	Tissue remodeling
Ppfibp1	PTPRF interacting protein, binding protein 1 (liprin beta 1)	NM_001170433	1.36	Tissue remodeling
Klf9	Kruppel-like factor 9	NM_010638	1.33	Tissue remodeling
Srgap1	SLIT-ROBO Rho GTPase activating protein 1	NM_001081037	1.33	Tissue remodeling
chr4:101010948-101032148_R		chr4:101010948-101032148_R	1.33	Unknown function
Pdgfra	Platelet derived growth factor receptor, alpha polypeptide	NM_011058	1.33	Tissue remodeling
Coro2b	Coronin, actin binding protein, 2B	NM_175484	1.32	Tissue remodeling
Fgfr1	Fibroblast growth factor receptor 1	NM_010206	1.29	Tissue remodeling
Rdm1	RAD52 motif 1	NM_025654	1.27	Other
Hipk3	Homeodomain interacting protein kinase 3	NM_010434	1.27	Other
Osbpl11	Oxysterol binding protein-like 11	NM_176840	1.25	Fatty acid metabolism
Larp6	La ribonucleoprotein domain family, member 6	NM_026235	1.23	Tissue remodeling
Ggt6	Gamma-glutamyltransferase 6	NM_027819	−1.96	Unknown function
Ccl19	Chemokine (C-C motif) ligand 19	NM_011888	−2.06	Inflammation
Scd2	Stearoyl-Coenzyme A desaturase 2	NM_009128	−2.13	Fatty acid metabolism
A_55_P2034320		A_55_P2034320	−2.21	Unknown function
Plch2	Phospholipase C, eta 2	NM_175556	−2.25	Other
Tuba1a	Tubulin, alpha 1A	NM_011653	−2.28	Tissue remodeling
Atp1a4	ATPase, Na+/K+ transporting, alpha 4 polypeptide	NM_013734	−2.31	Other
Gm6484	Predicted gene 6484	NM_001080940	−2.33	Fatty acid metabolism
D3Bwg0562e	DNA segment, Chr 3, Brigham & Women's Genetics 0562 expressed	NM_177664	−2.37	Other
Pla2g2e	Phospholipase A2, group IIE	NM_012044	−2.39	Fatty acid metabolism
Gys2	Glycogen synthase 2	NM_145572	−2.39	Carbohydrate metabolism/ insulin signaling
Serpina3m	Serine (or cysteine) peptidase inhibitor, clade A, member 3M	NM_009253	−2.39	Unknown function
Gm949	ADP-ribosylation factor-like 14 effector protein-like	NM_001033446	−2.44	Unknown function
Tekt1	Tektin 1	NM_011569	−2.78	Tissue remodeling
Ccl8	Chemokine (C-C motif) ligand 8	NM_021443	−2.86	Inflammation
Saa1	Serum amyloid A 1	NM_009117	−2.91	Inflammation
Saa3	Serum amyloid A 3	NM_011315	−3.49	Inflammation
S100a8	S100 calcium binding protein A8 (calgranulin A)	NM_013650	−4.31	Inflammation
Tph2	Tryptophan hydroxylase 2	NM_173391	−4.91	Other
Cck	Cholecystokinin	NM_031161	−8.00	Other

*Gene symbols are sorted based on fold change of the 22°C housed mice over 29°C housed mice*.

Pathway analysis of process networks and GO processes revealed that 8 of the top 10 process networks and 5 of the top 10 GO processes involved tissue remodeling related processes (Table 3). Furthermore, 2 of the top 10 process networks and 1 of the top 10 GO processes were related to inflammation.

**Table 3:** **Top 10 regulated process networks and gene ontology (GO) processes of eWAT in mice housed at 22°C compared to 29°C**.

	***p*****-value**	**FDR**	**Genes regulated**	**Total genes**
**PROCESS NETWORKS**				
1. Cytoskeleton_Cytoplasmic microtubules	2.96E-03	2.02E-01	7	115
2. Inflammation_IL-6 signaling	3.58E-03	2.02E-01	7	119
3. Development_Neurogenesis_Axonal guidance	4.85E-03	2.02E-01	10	230
4. Cell cycle_G0-G1	6.39E-03	2.02E-01	5	71
5. Cell adhesion_Attractive and repulsive receptors	8.60E-03	2.02E-01	8	175
6. Cytoskeleton_Spindle microtubules	9.46E-03	2.02E-01	6	109
7. Cytoskeleton_Regulation of cytoskeleton rearrangement	1.11E-02	2.03E-01	8	183
8. Cell adhesion_Leucocyte chemotaxis	2.07E-02	3.32E-01	8	205
9. Cell cycle_Mitosis	2.95E-02	4.19E-01	7	179
10. Cell cycle_Mitosis	3.35E-02	4.19E-01	7	184
**GO PROCESSES**				
1. Acute inflammatory response	7.73E-10	1.82E-06	13	122
2. Regulation of cell migration	8.22E-10	1.82E-06	30	754
3. Organic hydroxy compound metabolic process	1.55E-09	1.86E-06	27	636
4. Regulation of cellular component movement	2.02E-09	1.86E-06	32	881
5. Regulation of molecular function	2.32E-09	1.86E-06	72	3,333
6. Regulation of cell motility	2.90E-09	1.86E-06	30	796
7. Regulation of catalytic activity	2.93E-09	1.86E-06	63	2,735
8. Alcohol metabolic process	4.09E-09	2.27E-06	22	448
9. Regulation of locomotion	6.11E-09	2.93E-06	31	872
10. Response to external stimulus	6.61E-09	2.93E-06	62	2,725

*Based on unique genes with a false discovery rate (FDR) adjusted p < 0.1*.

Next, we assigned the significantly regulated genes with known functions, 93 of 125 genes, to biological processes. The most common processes related to tissue remodeling (34%), followed by inflammation (13%), fatty acid metabolism (9%), cholesterol and lipoprotein metabolism (8%), carbohydrate metabolism/insulin signaling (6%), and oxidative stress (5%) (Figure 4A). In the process with most regulated genes, tissue remodeling, the cytoskeleton genes such as tubulin-members alpha 1A (*Tuba1a*) and tubulin gamma 1 (*Tubg1*), mainly showed a lower expression in the 22°C housed mice (Figure 4B), which may be related to the observed decreased fat mass (and presumably reduced adipocyte size) in these mice compared to the 29°C housed control mice. Among cell differentiation/ growth genes, we see up regulation as well as down regulation, likely reflecting functional repositioning of the tissue.

The second most regulated process, inflammation, showed overall a marked decrease for 22°C housed mice. We specifically examined known key genes for pro-inflammatory M1 or anti-inflammatory M2 macrophages. Key genes of M1 macrophage inflammation, such as S100 calcium binding protein A8 (*S100a8*), and chemokine (C-C motif) ligand 8 (*Ccl8*) were significantly down regulated or showed a trend toward a down regulation in the 22°C housed mice, while M2 macrophage genes were not differentially regulated (Figure 4B). This suggests a decreased M1 over M2 ratio.

Subsequently, we verified transcript levels of genes involved in metabolism and analyzed the expression of known key genes involved in metabolism of eWAT. Expression of some key genes of carbohydrate metabolism showed significant differences between the two groups of mice. For example, *Gys2* expression was decreased, and *Pdk4* expression was increased in the mice housed at 22°C (Figure 4B), relating to the switch to fatty acid oxidation as measured by RER. In contrast to expectation, key genes involved in lipolysis and fatty acid oxidation, such as hormone sensitive lipase (*Lipe*), patatin-like phospholipase domain containing 2 (*Pnpla2*, a.k.a. *Atgl*), and *Cpt1a*, were mostly unaltered. Some genes involved in fatty acid metabolism did show changed transcript levels, such as increased *Pla2g2e* expression and nearly significantly decreased *Fasn* expression levels in eWAT in 22°C housed mice (Figure 4B).

Specific analysis of genes involved in browning showed that none of the known key genes, e.g., cell death-inducing DNA fragmentation factor, alpha subunit-like effector A (*Cidea*), and deiodinase iodothyronine, type II (*Dio2*) showed any changed expression. *Ucp1* remained below detection, indicating absence of browning occurring in eWAT of the 22°C housed mice (Figure 4B).

**Figure 6:**
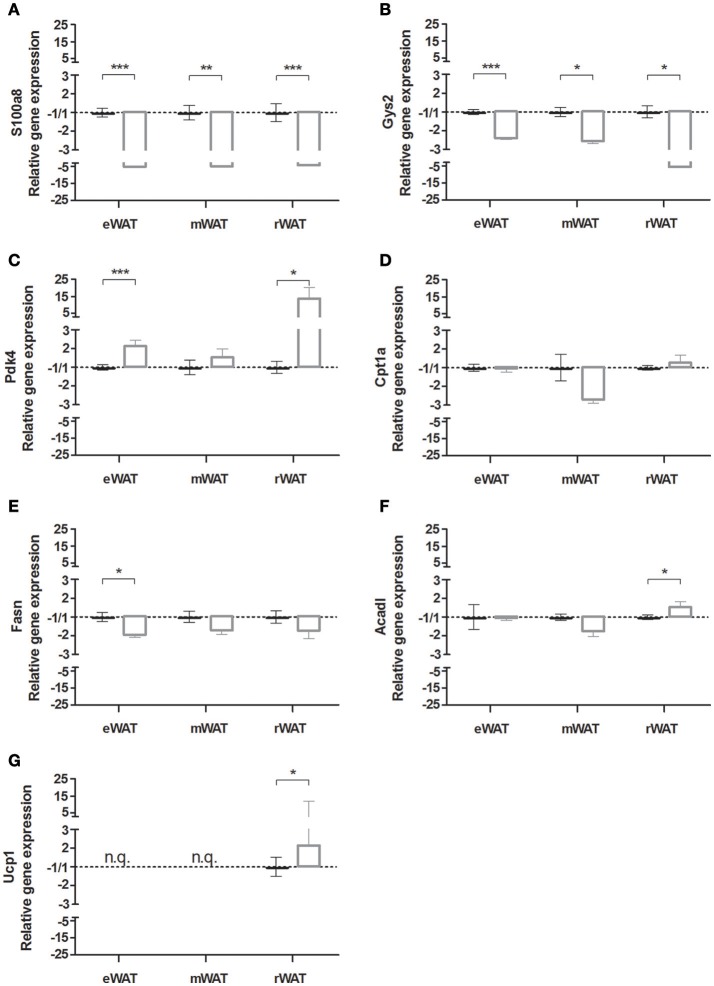
**Gene expression in white adipose tissue (WAT) depots of mice housed at either 29°C or 22°C**. Relative gene expression of **(A)** s100 calcium binding protein A8 (*s100a8*), **(B)** glycogen synthase 2 (*Gys2*), **(C)** pyruvate dehydrogenase kinase, isoenzyme 4 (*Pdk4*), **(D)** carnitine palmitoyltransferase 1a (*Cpt1a*), **(E)** fatty acid synthase (*Fasn*), **(F)** acyl-coenzyme A dehydrogenase long chain (*Acadl*), and **(G)** uncoupling protein 1 (*Ucp1*), of the 22°C vs. the 29°C housed mice in epididymal WAT (eWAT), mesenteric WAT (mWAT) and retroperitoneal WAT (rWAT). Data is presented as mean ± SEM (*n* = 4–10). Significant different values of the 22°C housed mice (white bar) compared to the 29°C housed mice (black bar) are indicated with ^*^p < 0.05, ^**^p < 0.01, and ^***^p < 0.001.

To extend our findings to other visceral WAT depots, expression of key marker genes was determined in eWAT, mWAT, and rWAT using RT-qPCR (Figure 6). The strong down regulation of inflammation, marked by the M1 macrophage associated gene *S100a8* was clearly observed in all three depots (Figure 6A). Similarly, the significant changes in the marker genes for carbohydrate metabolism were seen for all three depots: *Gys2* was down regulated in all three depots (Figure 6B), while *Pdk4* was up regulated, although not significant in mWAT (Figure 6C) Neglectable to small changes were seen for genes related to lipid metabolism; *Cpt1a* was unaltered in all three depots (Figure 6D), *Fasn* was significantly decreased only in eWAT (Figure 6E), and *Acadl* was significantly increased only in rWAT (Figure 6F). The browning marker *Ucp1* was not quantifiable in eWAT and mWAT, but appeared to be expressed and significantly increased in rWAT (Figure 6G).

## Discussion

In this study we showed that lowering the housing temperature of DIO mice from thermoneutrality to 22°C for 5 days resulted in upregulation of key thermogenic genes in BAT. Furthermore, body weight and total body fat were decreased, food intake was initially reduced but returned to amounts similar as seen before the switch to 22°C housing. Whole body EE, fatty acid oxidation, and physical activity were increased during the 5 days. This was associated with lower blood glucose, and serum insulin and leptin levels at 22°C housing. Moreover, most serum carnitine levels were increased, and serum FFA levels showed a trend toward increase, while most amino acids levels and serum TG levels were decreased, cumulative indicating altered substrate fluxes. Transcriptomics of eWAT revealed primarily downregulation of especially the cytoskeleton related genes within the tissue remodeling genes and a downregulation of M1 macrophages at 22°C vs. 29°C. Expression of some genes related to carbohydrate metabolism changed upon housing at 22°C, while expression of genes related to fatty acid metabolism was mostly unchanged. Furthermore, browning was increased at 22°C in only rWAT within 5 days, while not quantifiable in eWAT and mWAT. *Cck* and *Tph2* were the two most differently expressed genes due to the reduction in ambient temperature.

### Cold response in BAT

Increased gene expression levels of *Fgf21, Ppargc1a*, and *Ucp1* in BAT showed a similar response as found previously in cold (4–10°C) exposure experiments (Puigserver et al., 1998; Fisher et al., 2012; Shore et al., 2013; Ye et al., 2013), confirming that the relatively modest decrease in temperature from thermoneutrality to 22°C activates the adaptive thermogenic process in BAT in a similar way as during a more extreme cold exposure. In studies in which (near) thermoneutral temperatures (25–29°C) were lowered to room temperature (20–23°C), regulation of *Ucp1* levels appeared also to be related to the macronutrient composition of the diet consumed: mice fed high-fat diets increased *Ucp1* levels massively (this study and Rippe et al., 2000), while mice that consumed standard diets did not or only modestly increase *Ucp1* levels in BAT (Rippe et al., 2000; Uchida et al., 2010).

### Energy balance

EE was increased throughout the 5 days of decreased ambient temperature, in accordance with a recent study (Ravussin et al., 2012). In contrast to this study, which showed unchanged activity measurements after 44 days of room temperature housing compared to thermoneutrality, our study showed increased physical activity levels. Food intake was not increased after 5 days at 22°C, confirming previous findings (Rippe et al., 2000). Overall, this resulted in decreased body weight and fat mass within 5 days, supporting earlier findings in high fat-fed mice (Rippe et al., 2000; Ravussin et al., 2012). In our study, we observed a similar decrease in relative WAT mass in eWAT and rWAT. In contrast, mice fed a standard (low fat) diet showed absence of decreased body weight, due to increased food intake (Rippe et al., 2000; Uchida et al., 2010; Ravussin et al., 2012). This suggests that mice fed a standard (low fat) diet use increased dietary energy to compensate for the higher EE, while mice fed a high fat diet use internal fat stores in their body as energy source for the increased EE. Recently, it was shown that cold-exposed (4°C) DIO mice indeed increased the use of endogenous energy reserves, while mice with lower levels of obesity increased their food intake and used less of the endogenous energy reserves as energy source in the cold environment (Jaroslawska et al., 2015).

### Serum parameters

Increased energy demands due to higher EE in the cooler housed mice, combined with a lack of increased food intake, will deplete glycogen stores and energy supply will rely more on fatty acid usage (lower RER), obtained from internal fat stores, representing a negative energy balance. This was reflected in serum parameters. Although mice were exposed to a relative mild temperature reduction, both blood glucose levels and serum insulin levels were lower, suggesting an improved insulin sensitivity in these mice, similarly seen in cold exposed (4°C) mice (Bartelt et al., 2011). As a result, 22°C housed mice will have a relative lower insulin-suppressed lipolysis, thus increasing fatty acid oxidation (Schooneman et al., 2013), which is supported by the observed increased serum acylcarnitine/carnitine ratio combined with the decreased RER, and the increased *Pdk4* expression in eWAT and rWAT. Likewise, serum TG levels were decreased in the 22°C housed mice, supporting previous findings upon cold-exposure (4°C) in both obese and lean mice (Bartelt et al., 2011), likely caused by increased lipoprotein lipase activity in BAT upon noradrenalin stimulation (Carneheim et al., 1984). All serum amino acids tended to or were significantly decreased in the 22°C housed mice, potentially due to increased amino acid oxidation as seen previously in states of low energy intake such as prolonged fasting (Thompson et al., 2012). Lastly, serum leptin levels were greatly decreased in mice housed at 22°C. Cold exposure of 30 min at 4°C already reduced plasma leptin levels in humans (Pizon et al., 2009).

### WAT gene expression

Expression of genes involved in browning of eWAT was not induced by reducing housing temperature. Similarly, mice exposed to 10°C for 20 h showed induction of thermogenic genes in subcutaneous inguinal WAT (iWAT) but not in eWAT (Ye et al., 2013). *Ucp1* gene expression levels were likewise not induced in our study in mWAT; however, rWAT showed a cold-induced response with increased *Ucp1* expression. This corresponds to a relative resistance of eWAT and mWAT to browning, while rWAT could be considered a browning prone adipose tissue depot (Walden et al., 2012).

Markers for M1 (recruited) pro-inflammatory macrophages showed lower expression in eWAT in the mice housed at 22°C compared to 29°C, while M2 (resident) anti-inflammatory macrophage markers did not show a different expression between the two groups of mice. This effect seems similar in mWAT and rWAT based on the decreased *S100a8* gene expression in these WAT depots. Therefore, a shift toward a lower M1 over M2 ratio seems to occur in the WAT of 22°C housed mice. This shift has been associated with improved insulin sensitivity (as reviewed by Osborn and Olefsky, 2012). Similar to our study, decreased M1 and unchanged M2 marker expression were obtained in perivascular fat of ApoE^−/−^ mice housed at 22°C compared to 30°C; however, these mice did not show a difference in insulin resistance, but progression of atherosclerosis, a chronic inflammatory disease, was lower in the 22°C mice (Tian et al., 2016). A prolonged and/or more pronounced decrease in M1 macrophages might lead to increased insulin sensitivity. For example, switching mice from a HFD to a chow diet for 3 weeks changed the M1 macrophage phenotype to a less pro-inflammatory state, coincidental with an increased insulin sensitivity (Li et al., 2010), which agrees with the improved HOMA-IR we found in our study.

Overall, fatty acid metabolism hardly appeared to be affected when analyzed by transcript levels increased in visceral WAT of mice housed at 22°C. Similarly, lipolysis was not changed in WAT of female standard-fed mice housed for 24 h at either 22°C or 8°C (Shore et al., 2013). Because several serum parameters, e.g., carnitine levels, adiposity, and RER indicate increased fatty acid oxidation in 22°C housed mice, likely other WAT depots, such as subcutaneous WAT, or other metabolic organs, like liver and BAT, play a more prominent role than eWAT to adapt to the cooler ambient temperature.

Lower *Gys2* expression in the animals suggests that less glucose is stored as glycogen in eWAT, mWAT, and rWAT. Glycogen turnover has been shown also to play a role in adipose tissue in between carbohydrate and lipid metabolism (Markan et al., 2010), as well as linked to WAT inflammation in humans (Ceperuelo-Mallafre et al., 2016). Next to this, increased *Pdk4* levels might link to the lower glucose levels or reduced glycogen turnover, and indicate a decreased conversion of pyruvate in the carbohydrate oxidation pathway. Overall, the expression of these genes indicate that carbohydrate catabolism might be decreased in WAT of 22°C housed mice, which is in line with the lower RER.

### Cck and Tph2 expression in WAT

We unexpectedly observed expression of *Cck* in eWAT. CCK is known for its function in the gastrointestinal tract, where it is involved in activating gallbladder contraction, pancreatic enzyme secretion, and bile delivery to the duodenum; furthermore, CCK reduces meal size and increases behavioral satiety (for a review, see Moran and Kinzig, 2004). It is proposed that the main mechanism involved in inducing CCK-related satiety involves stimulation of vagal afferents located adjacent to enteroendocrine I cells, where CCK is produced (Larsson and Rehfeld, 1978; Moran and Kinzig, 2004). Herewith, information regarding energy status of ingested food is send directly from the gastrointestinal tract to the central nervous system, providing negative feedback leading to feeding inhibition.

The function of CCK in WAT is unknown. However, WAT seems to be innervated by the SNS (Bamshad et al., 1998; Bowers et al., 2004). Noradrenalin released from the nerve endings, e.g., as a result of cold exposure, can stimulate adrenergic receptors on the membrane of the adipocytes, influencing lipolysis in these cells (Brito et al., 2008). Furthermore, sensory afferents have been shown to originate from WAT, which might be interpreted as a direct feedback mechanism involved in adipose tissue status and regulation (Fishman and Dark, 1987; Bartness et al., 2010). It could be hypothesized that CCK released from adipocytes stimulates the sensory afferents in WAT, similarly to the stimulation of vagal afferents by CCK in the intestines, thereby involved in reporting energy status of WAT to the central nervous system. The striking decrease in *Cck* expression (top altered gene expression between the groups) in both eWAT and rWAT upon exposure to mild cold, as shown in our study, could thereby inform the brain of depleting energy storage in WAT. This seems to be reflected by the gradual increase in food intake during days 1–5. However, more research is needed to establish the role of sensory fibers in WAT and the potential function of CCK in this process.

Interestingly, the second most decreased transcript in the 22°C housed mice is *Tph2*. *Tph1* and *Tph2* are the rate limiting enzymes of serotonin production. *Tph1* was shown to be expressed peripherally, while *Tph2* was shown to induce central production of serotonin in neurons (Walther et al., 2003). Serotonin and CCK are both involved as signaling molecules in digestion of food and the regulation of food intake. Both serotonin and CCK have been shown to stimulate vagal afferent fibers, even interacting synergistically, which could result in a modulation of the signals from the gastrointestinal tract and thereby influencing the regulation of the central nervous system (Li et al., 2004). It was shown that Tph1^−/−^ mice probably have a higher sensitivity for β-adrenergic stimulation, which resulted in increased thermogenesis in BAT, and increased gene expression of browning-related genes in eWAT and iWAT compared to Tph1^+/+^ mice (Crane et al., 2015). This suggests that the impressively decreased *Tph2* expression in eWAT of 22°C housed mice might have a lowered serotonin production, resulting in browning and/ or lipolysis of WAT depots and increased BAT uncoupling as suggested by, i.e., *Ucp1* expression. Indeed, total fat mass and adiposity were significantly reduced.

## Conclusion

Housing at 29°C or switched to an ambient temperature of 22°C for 5 days induces metabolic changes in DIO mice. Mice housed at 22°C increased expression of thermogenic genes in BAT, increased whole body EE with concomitant increased activity and increased fatty acid to carbohydrate oxidation ratio compared to control thermoneutral housed mice, while food intake remained similar. Visceral adipose tissue, although being considered to be relatively resistant to thermogenesis-induced browning, showed adaptational changes to the reduction in temperature. The most pronounced different processes were a decrease in cytoskeleton regulation and a decreased M1 inflammatory over M2 anti-inflammatory markers of macrophages, accompanied by decreased circulating glucose and insulin levels, and an increased serum acylcarnitine/carnitine ratio. Concomitantly, *S100a8* gene expression was decreased in eWAT, mWAT, and rWAT. Expression of genes related to carbohydrate metabolism indicated decreased carbohydrate oxidation, while fatty acid oxidation was not increased in eWAT, mWAT, and rWAT. Browning was not induced in eWAT, and *Ucp1* levels were only expressed and induced in rWAT. Furthermore, we postulate that the lower WAT *Cck* and eWAT *Tph2* levels could provide neurosignaling of energy status in WAT to the brain. In total, we showed that visceral WAT metabolism is altered when switching DIO mice from a thermoneutral environment to the ambient temperature they are usually housed at.

## Author contributions

Data collection and analysis: animal study (IvdS, FH, JK, EvS), indirect calorimetry (IvdS, FH, JK, EvS), blood and tissue collection (IvdS, FH), serum parameters (IvdS, FH, JŠ, DF), RNA isolation (IvdS, FH), reverse-transcription quantitative real-time polymerase chain reaction (IvdS, FH), whole genome gene expression microarrays (IvdS, FH, EvS, JK). All authors were involved in study design, interpretation of the data, drafting the manuscript and approval of the final manuscript, and agree to be accountable for aspects investigated.

### Conflict of interest statement

The authors declare that the research was conducted in the absence of any commercial or financial relationships that could be construed as a potential conflict of interest.
