# Supplementary material for: Metabolic Response of Visceral White Adipose Tissue of Obese Mice Exposed for 5 Days to Human Room Temperature Compared to Mouse Thermoneutrality
Source: Front Physiol. 2017 Mar 23;8:179. doi: 10.3389/fphys.2017.00179 (PMC5362617; doi:10.3389/fphys.2017.00179)
Supplement: Supplementary file 1 [file SupplementaryTables1and2.pdf]

Supplementary table 1. Primer sequences used for RT-qPCR.

Genes	Full name	Forward primer sequence (5' --> 3')	Reverse primer sequence (5' --> 3')	Tissue	Annealing temperature (ºC)
Fgf21	fibroblast growth factor 21	AGATCAGGGAGGATGGAACA	TCAAAGTGAGGCGATCCATA	BAT	57
Ppargc1a	peroxisome proliferative activated receptor, gamma, coactivator 1 alpha	CCCTGCCATTGTTAAGACC	TGCTGCTGTTCCTGTTTTC	BAT	60
Ucp1	uncoupling protein 1	CCGAAGTGCAACCCACCATGGG	CCTTCACCTTGGATCTGAAGGCGGA	BAT/WAT	60
Cck	cholecystokinin	ACTGCTAGCGCGATACATCC	CATCCAGCCCATGTAGTCCC	WAT	58
Tph2	tryptophan hydroxylase 2	TACACCCCGGAACCAGATAC	CAAAGGATTTCACACACGCC	WAT	58
Acadl	acyl-Coenzyme A dehydrogenase, long-chain	TGCACACATACAGACGGTGCAGC	GCAGAACCGGAGTCCAGACGT	WAT	60
Cpt1a	carnitine palmitoyltransferase 1a	AAAGATCAATCGGACCCTAGACA	CAGCGAGTAGCGCATAGTCA	WAT	57
Fasn	fatty acid synthase	AGTTAGAGCAGGACAAGCCCAAG	GTGCAGAGCTGTGCTCCTGA	WAT	58
Gys2	glycogen synthase 2	TTGTCGGTGACATCCCTTGG	TTGGCCTTGGTCTGGATCAC	WAT	62
Pdk4	pyruvate dehydrogenase kinase, isoenzyme 4	ACTCCACTGCTCCAACACCTG	TGATAGCGTCTGTCCCATAACCTG	WAT	60
S100a8	S100 calcium binding protein A8 (calgranulin A)	ACTTCGAGGAGTTCCTTGCG	TGCTACTCCTTGTGGCTGTC	WAT	60
Reference genes					
B2m	beta-2 microglobulin	CCCCACTGAGACTGATACATACGC	AGAAACTGGATTTGTAATTAAGCAGGTTC	BAT/WAT	60
Canx	calnexin	GCAGCGACCTATGATTGACAACC	GCTCCAAACCAATAGCACTGAAAGG	BAT	58
Rps15	ribosomal protein S15	CGGAGATGGTGGGTAGCATGG	ACGGGTTTGTAGGTGATGGAGAAC	WAT	60

Supplementary table 2. Microarray pathway analysis of gene profiles of eWAT of 22ºC housed mice compared to 29ºC housed mice.

Gene symbol	Gene name	Systematic name	FC	P
**Most changed genes**				
Cck	cholecystokinin	NM_031161	-8.00	0.015
Tph2	tryptophan hydroxylase 2	NM_173391	-4.91	0.011
**Browning**				
Dio2	deiodinase, iodothyronine, type II	NM_010050	-1.51	0.289
Prdm16	PR domain containing 16	NM_027504	1.03	0.985
Cidea	cell death-inducing DNA fragmentation factor, alpha subunit-like effector A	NM_007702	1.22	0.911
Ucp1	uncoupling protein 1 (mitochondrial, proton carrier)	NM_009463	below detection	
**Cytoskeleton**				
Tekt1	tektin 1	NM_011569	-2.78	0.018
Tuba1a	tubulin, alpha 1A	NM_011653	-2.27	0.012
Fam110c	family with sequence similarity 110, member C	NM_027828	-1.84	0.013
Tuba1b	tubulin, alpha 1B	NM_011654	-1.54	0.012
Tuba4a	tubulin, alpha 4A	NM_009447	-1.53	0.003
Pkp1	plakophilin 1	NM_019645	-1.51	0.036
Palld	palladin, cytoskeletal associated protein	NM_001081390	-1.37	0.014
Traf4	TNF receptor associated factor 4	NM_009423	-1.33	0.023
Capza1	capping protein (actin filament) muscle Z-line, alpha 1	NM_009797	1.11	0.034
Map1lc3b	microtubule-associated protein 1 light chain 3 beta	NM_026160	1.15	0.037
Coro2b	coronin, actin binding protein, 2B	NM_175484	1.32	0.040
**Cell migration/ growth**				
Igfals	insulin-like growth factor binding protein, acid labile subunit	NM_008340	-1.94	0.019
Insig1	insulin induced gene 1	NM_153526	-1.77	0.018
Cspg4	chondroitin sulfate proteoglycan 4	NM_139001	-1.61	0.012
Cdkn2b	cyclin-dependent kinase inhibitor 2B (p15, inhibits CDK4)	NM_007670	-1.52	0.018
Setd8	SET domain containing (lysine methyltransferase) 8	NM_030241	-1.43	0.024
Hdac9	histone deacetylase 9	NM_024124	-1.42	0.018
E2f2	E2F transcription factor 2	NM_177733	-1.41	0.027
Parm1	prostate androgen-regulated mucin-like protein 1	NM_145562	-1.36	0.040
Igf1	insulin-like growth factor 1	NM_010512	-1.28	0.012
Plxnd1	plexin D1	NM_026376	-1.28	0.015
Dph6	diphthamine biosynthesis 6	NM_025675	-1.19	0.024
Zfp467	zinc finger protein 467	NM_001085417	1.22	0.042
Klhl21	kelch-like 21	NM_001033352	1.22	0.024
Larp6	La ribonucleoprotein domain family, member 6	NM_026235	1.23	0.008
Fgfr1	fibroblast growth factor receptor 1	NM_010206	1.29	0.048
Pdgfra	platelet derived growth factor receptor, alpha polypeptide	NM_011058	1.33	0.026
Srgap1	SLIT-ROBO Rho GTPase activating protein 1	NM_001081037	1.33	0.018
Klf9	Kruppel-like factor 9	NM_010638	1.33	0.042
Ppfibp1	PTPRF interacting protein, binding protein 1 (liprin beta 1)	NM_001170433	1.36	0.029
Antxr1	anthrax toxin receptor 1	NM_054041	1.37	0.008
Twist2	twist basic helix-loop-helix transcription factor 2	NM_007855	1.42	0.002
Thbs2	thrombospondin 2	NM_011581	1.60	0.040
**Inflammation**				
Hp	haptoglobin	NM_017370	-1.90	0.027
Slc25a1	solute carrier family 25 (mitochondrial carrier, citrate transporter), member 1	NM_153150	-1.54	0.045
Tm7sf2	transmembrane 7 superfamily member 2	NM_028454	-1.38	0.013
Rab7l1	RAB29, member RAS oncogene family	NM_144875	-1.30	0.037
Twist2	twist basic helix-loop-helix transcription factor 2	NM_007855	1.42	0.002
Vnn1	vanin 1	NM_011704	2.03	0.019

Gene symbol	Gene name	Systematic name	FC	P
**Inflammation: M1 RNA markers**				
S100a8	S100 calcium binding protein A8 (calgranulin A)	NM_013650	-4.31	0.002
Saa3	serum amyloid A 3	NM_011315	-3.49	0.010
Saa1	serum amyloid A 1	NM_009117	-2.91	0.012
Ccl8	chemokine (C-C motif) ligand 8	NM_021443	-2.86	0.002
Cx3cr1	chemokine (C-X3-C motif) receptor 1	NM_009987	-2.40	0.550
Ccl19	chemokine (C-C motif) ligand 19	NM_011888	-2.06	0.008
Ccr2	chemokine (C-C motif) receptor 2	NM_009915	-1.55	0.111
Ccl2	chemokine (C-C motif) ligand 2	NM_011333	-1.48	0.366
Il1b	interleukin 1 beta	NM_008361	-1.38	0.304
Il6	interleukin 6	NM_031168	-1.05	0.968
**Inflammation: M2 RNA markers**				
Mrc1	mannose receptor, C type 1	NM_008625	-1.17	0.844
Clec10a	C-type lectin domain family 10, member A	NM_010796	-1.12	0.906
Cd163	CD163 antigen	NM_001170395	-1.06	0.980
Arg1	arginase, liver	NM_007482	-1.01	0.997
Retnla	resistin like alpha	NM_020509	1.12	0.988
**Glucose metabolism**				
Gys2	glycogen synthase 2	NM_145572	-2.22	0.002
Grb14	growth factor receptor bound protein 14	NM_016719	-1.39	0.032
Glyctk	glycerate kinase	NM_174846	-1.38	0.036
Alx3	aristaless-like homeobox 3	NM_007441	-1.35	0.015
Acly	ATP citrate lyase	NM_001199296	-1.22	0.433
Nudt14	nudix (nucleoside diphosphate linked moiety X)-type motif 14	NM_025399	-1.22	0.024
Slc2a1	solute carrier family 2 (facilitated glucose transporter), member 1	NM_011400	-1.00	0.999
Pdk4	pyruvate dehydrogenase kinase, isoenzyme 4	NM_013743	2.14	0.015
**Fatty acid metabolism/beta oxidation**				
Gm6484	angiopoietin-like 8	NM_001080940	-2.33	0.009
Scd2	stearoyl-Coenzyme A desaturase 2	NM_009128	-2.13	0.038
Pla2g2e	phospholipase A2, group IIE	NM_012044	-2.05	0.008
Fasn	fatty acid synthase	NM_007988	-1.86	0.119
Slc25a1	solute carrier family 25 (mitochondrial carrier, citrate transporter), member 1	NM_153150	-1.54	0.045
Cyb5b	cytochrome b5 type B	NM_025558	-1.53	0.028
Cpt1a	carnitine palmitoyltransferase 1a, liver	NM_013495	-1.44	0.914
Rab32	RAB32, member RAS oncogene family	NM_026405	-1.35	0.045
Acly	ATP citrate lyase	NM_001199296	-1.22	0.433
Dgat1	diacylglycerol O-acyltransferase 1	NM_010046	-1.05	0.983
Cpt2	carnitine palmitoyltransferase 2	NM_009949	-1.02	0.993
Plin2	perilipin 2	NM_007408	-1.01	0.997
Plin3	perilipin 3	NM_025836	-1.01	0.996
Mgat1	mannoside acetylglucosaminyltransferase 1	NM_001110149	1.01	0.993
Slc22a5	solute carrier family 22 (organic cation transporter), member 5	NM_011396	1.03	0.978
Acox1	acyl-Coenzyme A oxidase 1, palmitoyl	NM_015729	1.03	0.988
Acadm	acyl-Coenzyme A dehydrogenase, medium chain	NM_007382	1.08	0.930
Plin1	perilipin 1	NM_001113471	1.08	0.979
Lipe	lipase, hormone sensitive	NM_010719	1.17	0.925
Cd36	CD36 antigen	NM_001159556	1.19	0.627
Plin4	perilipin 4	NM_020568	1.22	0.930
Osbpl11	oxysterol binding protein-like 11	NM_176840	1.25	0.024
Fabp4	fatty acid binding protein 4, adipocyte	NM_024406	1.27	0.813
Pnpla2	patatin-like phospholipase domain containing 2	NM_001163689	1.34	0.385
Plin5	perilipin 5	NM_025874	1.36	0.485
Slc27a1	solute carrier family 27 (fatty acid transporter), member 1	NM_011977	1.39	0.028

Expression of the 2 strongest regulated genes, and key genes for browning, tissue remodelling, inflammation, and metabolism. Genes are ranked based on FC per subgroup. Underlined genes are key markers for the assigned processes. FC = fold change, P = Benjamini-Hochberg false discovery rate-adjusted p-value of Student's *t*-test.
